# Oral Liposomal Iron Versus Injectable Iron Sucrose for Anemia Treatment in Non-dialysis Chronic Kidney Disease Patients: A Non-inferiority Study

**DOI:** 10.7759/cureus.70114

**Published:** 2024-09-24

**Authors:** Soufiane Bengelloun Zahr, Yassine Allata, Mouna El Mansoury, Basmat Amal Chouhani, Nadia Kabbali, Ghita El Bardai, Tarik Sqalli Houssaini

**Affiliations:** 1 Nephrology, Dialysis, and Transplantation, Hassan II University Hospital, Fez, MAR; 2 Laboratory of Epidemiology and Health Science Research, Faculty of Medicine, Sidi Mohammed Ben Abdellah University, Fez, MAR

**Keywords:** chronic kidney disease, ferritin, hemoglobin, intravenous iron sucrose, liposomal iron, transferrin saturation

## Abstract

Introduction

Anemia is a prevalent and persistent complication in chronic kidney disease (CKD), particularly in advanced stages, contributing to the deterioration of renal function and diminishing patients' quality of life. Iron supplementation constitutes a cornerstone of anemia management in this population. Among various iron formulations, liposomal iron has emerged as a promising option due to its enhanced efficacy in replenishing iron reserves and improved tolerability.

Objective

This study aims to assess the comparative effects of intravenous and liposomal oral iron on hemoglobin levels in non-dialysis CKD patients. Additionally, it seeks to evaluate the rate of hemoglobin correction, iron reserve status during treatment, and therapeutic tolerance to these interventions.

Materials and methods

A randomized controlled trial enrolled CKD patients (stages 3-5, not on dialysis) with iron deficiency anemia (hemoglobin ≤ 12 g/dL, ferritin ≤ 100 ng/mL, transferrin saturation ≤ 25%). Participants were allocated to receive either daily oral liposomal iron (Group OS) at a dosage of 30 mg or intravenous iron-hydroxide sucrose complex weekly (Group IV) for three months. Follow-up extended through the treatment phase and two months post-withdrawal.

Results

Thirty-one CKD patients were randomized into two groups: 14 received intravenous iron (IV group) and 17 received oral iron (OS group). After excluding four patients, the final cohort comprised 27 individuals (IV group: n=13, OS group: n=14). Both iron treatments resulted in progressive hemoglobin increases, with the IV group showing a mean increase of 14.65% (p=0.049) compared to 4.78% (p=0.003) in the OS group. Secondary analysis revealed significant increases in ferritin levels (p<0.001) and transferrin saturation (TSAT) levels (p=0.031) in the IV group. Post-treatment follow-up demonstrated stable hemoglobin levels in the OS group and a consistent increase in ferritin levels in the IV group. Adverse reactions predominantly included hypotension in the IV group (4 (30.7%)) and constipation in the OS group (4 (28.4%)).

Discussion and conclusion

Anemia remains a significant challenge in CKD patients. Our study compares oral liposomal iron to injectable iron for anemia treatment, aiming to minimize hospitalizations for iron infusion, preserve venous capital, and mitigate potential harmful side effects. We found oral liposomal iron to be a safe and effective option for correcting anemia in non-dialysis CKD patients, albeit with lower efficacy in replenishing iron stores compared to IV iron. Comparative analysis with similar studies supports the non-inferiority of oral liposomal iron, although IV iron retains superiority in replenishing iron reserves.

## Introduction

The last two and a half decades have seen erythropoiesis-stimulating agents (ESAs) and iron therapy at the forefront of managing anemia in chronic kidney disease (CKD) patients. While ESAs have demonstrated significant efficacy in alleviating anemia in this context, recent large-scale randomized controlled trials in both non-dialysis and dialysis CKD patients have highlighted their limitations [1].

Attempts to normalize hemoglobin (Hb) levels with ESAs have shown no significant cardiovascular benefits and have been associated with increased risks of adverse events, such as stroke and venous thromboembolism [2]. Consequently, there has been a reduction in ESA prescriptions, an increase in blood transfusions, and a notable rise in the use of iron therapy due to its role in addressing hypo responsiveness to ESAs.

Anemia remains a common complication in CKD, significantly impacting cardiovascular health and quality of life. While a deficit in renal erythropoietin production is a primary cause, iron deficiency plays a pivotal role in the genesis of CKD-related anemia. Iron deficiency, whether absolute or functional, alongside an inflammatory block often seen in CKD patients, accounts for the main reasons behind hypo responsiveness to ESAs [3].

The recent shift from ESA-centered treatment to iron therapy has prompted debates regarding the ideal route of iron administration, particularly in non-dialysis CKD patients. Despite the benefits of oral iron, including cost-effectiveness and ease of administration, its use remains limited due to poor gastrointestinal absorption and high rates of adverse events. Conversely, concerns about intravenous (IV) iron revolve around potential kidney damage, infections, atherosclerosis promotion, and other adverse reactions.

Given these considerations, our study aims to compare the efficacy of oral liposomal iron versus IV iron in treating anemia in non-dialysis CKD patients in terms of iron status during treatment, Hb levels, speed of Hb correction, and therapeutic tolerance.

## Materials and methods

Study design

We conducted a randomized controlled trial in the Nephrology, Dialysis, and Transplantation Department of the University Hospital Center Hassan II of Fes. We screened 81 consecutive patients with CKD (stages 3-5) from July 2021 to July 2023. In accordance with the International Committee of Medical Journal Editors (ICMJE) guidelines, our trial is registered at ClinicalTrials.gov under the ID NCT06556134.

Inclusion criteria

Participants were required to be over 18 years of age, with an estimated glomerular filtration rate (eGFR) of ≤60 mL/min/1.73 m² (using the Modification of Diet in Renal Disease equation, MDRD), Hb levels ≤12 g/dL, ferritin levels ≤100 ng/mL, transferrin saturation (TSAT) ≤25%, and parathyroid hormone (PTH) serum levels between 30 and 300 pg/mL.

Exclusion and withdrawal criteria

Patients were excluded if they had inflammatory or infectious diseases with C-reactive protein (CRP) levels ≥6 mg/L at the study's onset, had undergone surgical interventions within the last three months, or had hematological disorders, including recent bleeding or blood transfusions (within the past six months). Additional exclusion criteria included malignancies, ongoing immunosuppressive treatments, severe malnutrition (defined as a BMI <20 kg/m², hypoalbuminemia <32 g/L with normal CRP levels, or weight loss >5% in one month or >7.5% in three months), major cardiovascular disease (myocardial infarction, chronic heart failure, stroke), chronic alcohol abuse, known hepatitis B or C infection, pregnancy or lactation, and the need to start dialysis.

Withdrawal from the study was mandated in cases of severe anemia requiring urgent blood transfusion, non-adherence to the protocol, or withdrawal of consent.

Ethics committee approval

The trial received approval from Comite D’ethique Hospitalo-Universitaire Fes (approval number: 01/20) and strictly adhered to the principles outlined in the Declaration of Helsinki.

Informed consent

In our study, the process of obtaining informed consent ensured that participants fully understood the nature and objectives of the research. Prior to consenting, participants received a comprehensive explanation of the study's purpose, design, and hypothesis. This information was presented in a clear and understandable manner to facilitate informed decision-making. To further enhance comprehension, participants were provided with an information form that outlined all relevant details, and this document was made available in both French and Arabic.

The bilingual information form aimed to cater to the diverse linguistic preferences of the study participants, ensuring that each individual had access to the necessary information in their preferred language. Following the detailed explanation of the study, participants were provided with a consent form in both French and Arabic languages and were encouraged to sign the consent form in their preferred language, ensuring that they fully comprehended the information presented. The consent form explicitly outlined all their rights, including the crucial right to withdraw from the study at any time without facing any repercussions.

This approach adhered to ethical standards, prioritizing transparency and respect for participant autonomy throughout the informed consent process.

Screening and randomization

We screened 81 patients in accordance with our inclusion and exclusion criteria. The recruited patients underwent historical and clinical assessments and discontinued any oral iron supplementation that was not part of the study. Subsequently, the participants were randomized into the treatment phase at baseline (referred to as M0). The randomization process was generated by a statistician, and the allocation was concealed through sequentially numbered, sealed envelopes. These envelopes were opened by staff members who were not involved in any step of patient care.

Study protocol

The first group (Group IV) received IV iron-hydroxide sucrose complex (marketed as Fermed® 100 mg), administered at a dose of 100 mg, diluted in 250 mL of normal saline, and infused weekly for three months. In contrast, the second group (Group OS) received one oral capsule per day containing 30 mg of pyrophosphate liposomal iron and 70 mg of ascorbic acid (marketed as Lisofer® 30 mg) for the same three-month duration. Before receiving the iron treatment, subjects underwent clinical evaluations, which were repeated immediately after the iron infusion, as well as at 30- and 60-minute post-infusion. Laboratory tests were conducted monthly, starting at baseline (M0) and during all follow-up visits at months 1 (M1), 2 (M2), and 3 (M3), as well as two months post-treatment discontinuation (M4 and M5). These tests included Hb levels, ferritin serum levels, TSAT, CRP, and serum albumin levels. A detailed operating sheet was prepared for each patient to collect and monitor comprehensive data throughout the study period. This sheet recorded clinical and laboratory information, including baseline clinical details such as weight, recent infections or hospitalizations, eGFR, CKD stage, and specific renal diseases, if present. Baseline laboratory data such as Hb, ferritin, TSAT, PTH, CRP, albumin, calcium, and phosphate levels were also recorded. Information about the concurrent use of ESAs, angiotensin receptor blockers (ARBs), or angiotensin-converting enzyme inhibitors (ACE-Is) was documented to assess potential confounding factors. Throughout the study, the operating sheet facilitated ongoing patient monitoring, tracking clinical and laboratory changes, and documenting any observed side effects or adverse events. The doses of ACE-Is/ARBs and ESAs were managed by the clinician according to personal habits, independently of the study. During the study period, iron therapy was suspended if TSAT exceeded 50% or if ferritin levels were >800 ng/mL.

Compliance and adverse effects evaluations

Patients in both groups were followed up monthly for compliance (Questionnaire A) and potential adverse effects and their severity (Questionnaire B). Questionnaire B specifically asked patients to quantify (none, somewhat/occasionally, a lot/often) the frequency of various symptoms, including gastrointestinal symptoms (nausea, vomiting, abdominal pain, bloating, constipation (<1 bowel movement per two days), diarrhea (>3 bowel movements per day)), immune system symptoms (hypersensitivity, angioedema, anaphylactic reactions), nervous system symptoms (dysgeusia, headaches, vertigo, paresthesia, hypoesthesia, syncope, drowsiness, confusion, decreased level of consciousness, anxiety, tremor), cardiac symptoms (bradycardia, tachycardia, palpitations, chest pain), vascular symptoms (hypotension, hypertension, flushing, thrombophlebitis, circulatory collapse), respiratory symptoms (dyspnea, bronchospasm), urinary symptoms (chromaturia), dermatological symptoms (pruritus, rash, urticaria, erythema), musculoskeletal and systemic symptoms (muscle spasms, myalgias, arthralgias, back pain), and general symptoms, as well as others related to the administration site (fever, chills, fatigue, peripheral edema, hyperhidrosis, cold sweat, malaise, pallor, flu symptoms, reactions at the injection/perfusion site).

Statistical analysis

Data collected were tabulated in a Microsoft Excel sheet (Microsoft Corporation, Redmond, Washington). The means and standard deviations of the measurements per group were used for statistical analysis using IBM SPSS Statistics for Windows, Version 26 (Released 2019; IBM Corp., Armonk, New York). The differences between the two groups were assessed using the Friedman test, a non-parametric statistical test, with the p-value set at < 0.05.

## Results

Baseline data

**Figure 1:**
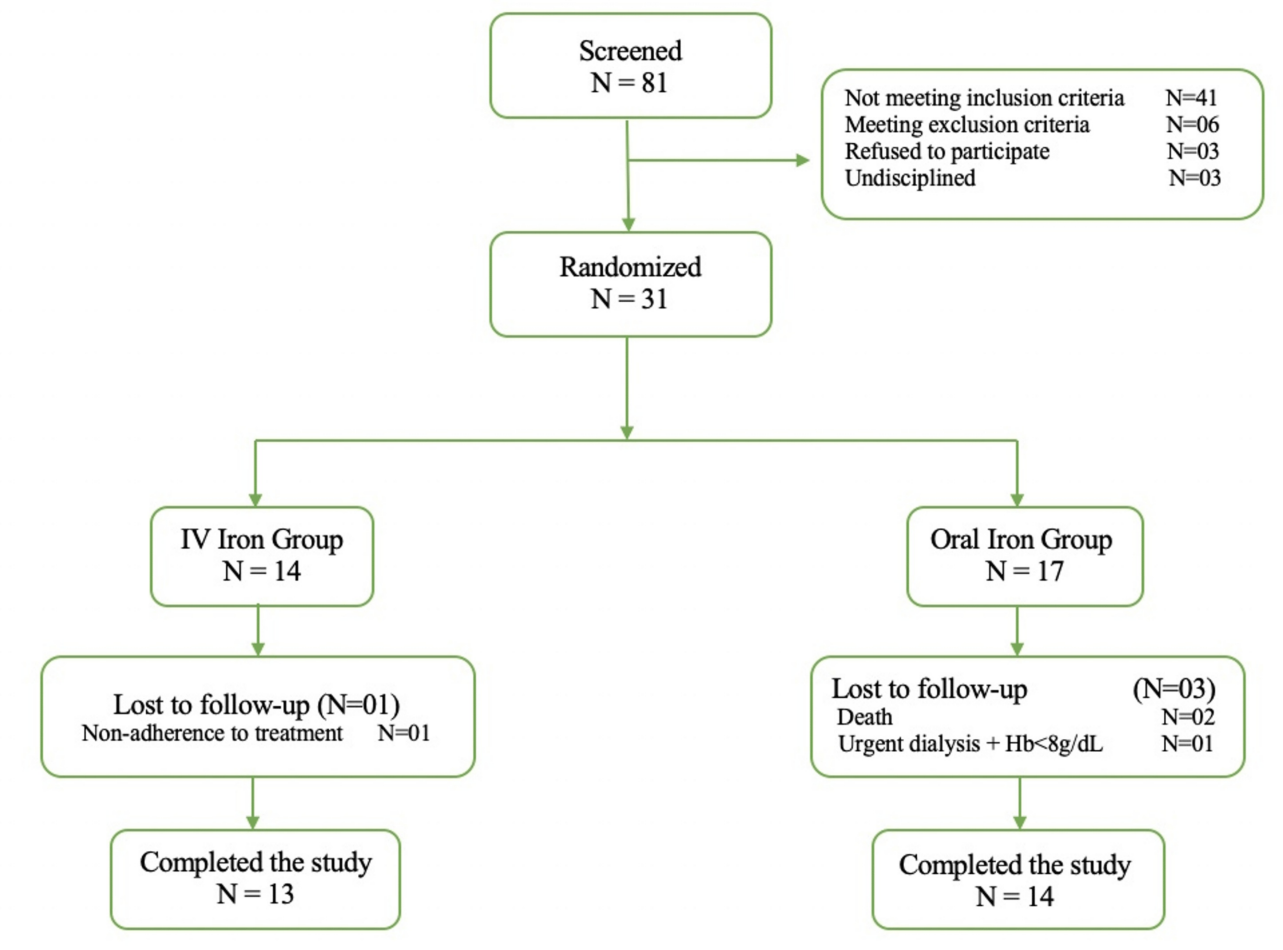
A flowchart illustrating patient recruitment and disposition in the study Hb: hemoglobin

As depicted in Figure 1, out of the initial pool of 81 patients assessed for eligibility, 31 individuals were randomized into two distinct treatment groups: 14 were assigned to the IV iron group (IV group), while 17 were allocated to the oral iron group (OS group). Four patients were excluded from the study for various reasons: one individual from the IV group due to non-adherence to treatment, and three individuals from the OS group (two deaths and one requiring urgent dialysis with a Hb level <8 g/dL). Consequently, the subsequent statistical analysis was conducted on a final cohort of 27 patients, comprising 13 individuals in the IV group and 14 in the OS group. During the study period, one individual's death was attributed to what appeared to be a myocardial infarction, while the cause of death for the second participant remained undetermined.

The primary analysis of each group's baseline data compares demographics, anthropometric measures, renal function, renin-angiotensin-aldosterone system (RAAS) inhibitor use, and variances in renal disease between the OS and IV groups. The characteristics of these patients are summarized in Tables 1, 2. Baseline laboratory data are also presented in Table 3. No significant differences were detected between the two groups in the key laboratory parameters, including those related to anemia.

**Table 1:** Demographic characteristics of the two groups under study eGFR: estimated glomerular filtration rate, RAASi: renin-angiotensin-aldosterone system inhibitors, SD: standard deviation.

Variables	IV Iron Group (N=13)	Oral Iron Group (N=14)
Male	6 (46.2%)	4 (28.6%)
Female	7 (53.8%)	10 (71.4%)
Age (years) ± SD	55.79 ± 18.53	59.29 ± 14.34
Weight (kg) ± SD	69.64 ± 11.1	73.29 ± 12.99
Body mass index (kg/m²) ± SD	26.08 ± 4.76	28.48 ± 6.07
Creatinine (mg/L) ± SD	26.21 ± 10.54	38.29 ± 19.64
eGFR (mL/min/1.73 m²) ± SD	25.64 ± 8.05	18.82 ± 8.91
RAASi use	9 (69.2%)	12 (85.7%)

**Table 2:** Variances in renal diseases in the two groups under study

Renal diseases	IV Iron Group (N=13)	Oral Iron Group (N=14)
Diabetic kidney disease	8 (61.5%)	7 (50%)
Nephroangiosclerosis	-	1 (7.15%)
Tubulointerstitial nephritis	1 (7.7%)	1 (7.15%)
Urological disorders	1 (7.7%)	1 (7.15%)
Others	3 (23.1%)	1 (7.15%)
Indeterminate	-	3 (21.4%)

**Table 3:** Laboratory data of the patients in the two groups at baseline The values are expressed in means ± SD. TSAT: transferrin saturation, PTH: parathyroid hormone, CRP: C-reactive protein, SD: standard deviation.

Parameters	Oral Iron Group (N = 14)	IV Iron Group (N = 13)
Hb (g/dL)	9.82 ± 1.21	10.03 ± 0.66
Ferritin (ng/mL)	64.45 ± 37.41	60.71 ± 35.89
TSAT (%)	12.89 ± 9.3	11.33 ± 3.05
Serum iron (mg/L)	0.54 ± 0.25	0.5 ± 0.12
Vitamin B12 (pg/mL)	378 ± 145	409 ± 137
Vitamin B9 (ng/mL)	6.7 ± 2.8	6.1 ± 3.2
Albumin (g/L)	39.9 ± 2.11	39.71 ± 4.3
PTH (pg/mL)	256 ± 96	298 ± 106
CRP (mg/L)	3.17 ± 2.63	3.8 ± 2.2

Follow-up data

Both iron treatments were associated with a progressive increase in Hb levels (M5 versus respective M0), although to varying degrees. By the end of the treatment period, the mean increases in Hb levels (M5 versus M0) were 14.65% for the IV group and 4.78% for the OS group.

As shown in Tables 4, 5, the secondary analysis examines follow-up data to evaluate the effects of both oral and IV iron treatments on various laboratory parameters, including Hb levels, serum iron, TSAT, ferritin, hsCRP, and albumin.

**Table 4:** Clinical and laboratory data in the OS group throughout the study The values are expressed in means ± SD. Hb: hemoglobin, TSAT: transferrin saturation, CRP: C-reactive protein.

Parameters	Oral Iron Group (N=14)							
	M0	M1	M2	M3	M4	M5	p-value	Chi-Square Value
Hb (g/dL)	9.82 ± 1.21	9.88 ± 1.15	9.94 ± 1.40	10.04 ± 1.24	10.32 ± 1,46	10.29 ± 1.61	0.003	17.68
Ferritin (ng/mL)	64.45 ± 37.41	93.72 ± 66.68	95.65 ± 33.99	97.45 ± 78.91	98.36 ± 61.81	92.90 ± 64.60	0.399	5.14
Serum iron (mg/L)	0.54 ± 0.25	0.70 ± 0.15	0.60 ± 0.16	0.56 ± 0.14	0.57 ± 0.11	0.61 ± 0.13	0.35	5.57
TSAT (%)	12.89 ± 9.3	13.12 ± 9.01	13.56 ± 4.90	14.28 ± 4.74	14.52 ± 6.78	14.19 ± 5.12	0.057	10.72
Albumin (g/L)	39.9 ± 2.11	40.18 ± 2.13	41 ± 2.79	40.18 ± 2.31	39.45 ± 2,46	40 ± 1.84	0.436	4.83
CRP (mg/L)	3.17 ± 2,63	6.77 ± 12.27	3.35 ± 2.52	2.78 ± 2.28	3.35 ± 2.22	3.37 ± 1.93	0.839	2.07

**Table 5:** Clinical and laboratory data in the IV group throughout the study The values are expressed in means ± SD. Hb: hemoglobin, TSAT: transferrin saturation, CRP: C-reactive protein.

Parameters	IV Iron Group (N=13)							
	M0	M1	M2	M3	M4	M5	p-value	Chi-Square Value
Hb (g/dL)	10.03 ± 0.66	10.86 ± 0.68	11.57 ± 0.60	11.62 ± 0.80	11.65 ± 0.85	11.50 ± 0.96	0.049	11.11
Ferritin (ng/mL)	60.71 ± 35.89	189.85 ± 116.57	233.28± 148.44	242.85 ± 134.02	282.85 ± 100.35	289.42 ± 103.13	0.0001	23.98
Serum iron (mg/L)	0.50 ± 0.12	0.66 ± 0.13	0.74± 0.39	0.83 ± 0.32	0.67 ± 0.14	0.80 ± 0.23	0.099	9.26
TSAT (%)	11.33 ± 3.05	14.00 ± 2.00	15.33 ± 1.52	19.33 ± 7.57	19.66 ± 6.42	19.45 ± 6.92	0.031	12.28
Albumin (g/L)	39.71 ± 4.30	39.00 ± 4.12	38.85 ± 5.75	39.4 ± 3.97	39.71 ± 5.43	38.85 ± 4.63	0.696	3.02
CRP (mg/L)	3.85 ± 2.19	6.44 ± 9.56	2.94 ± 1.86	2.64 ± 1.03	2.45 ± 1.59	2.48 ± 1.46	0.494	4.39

**Figure 2:**
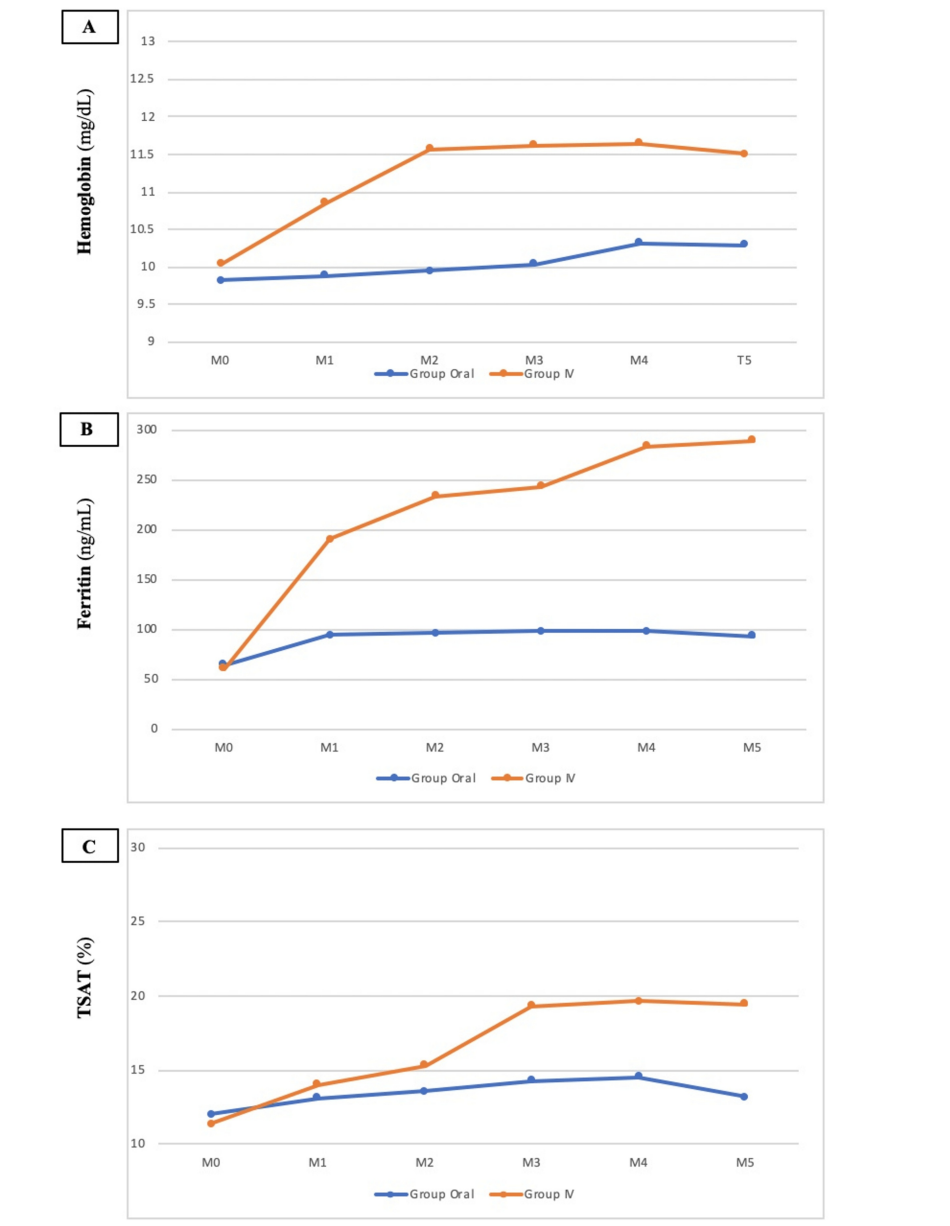
Effects of IV (Group IV, red lines) and oral liposomal iron (Group OS, blue lines) on hemoglobin (A), ferritin (B), and TSAT (C) throughout the study Data are expressed as means. M0, baseline levels; M1, M2, and M3, values after one, two, and three months of follow-up with both drugs; M4, one month after drug withdrawal; M5, two months after drug withdrawal. TSAT: transferrin saturation.

In the oral treatment group (Group OS), hemoglobin (Hb) levels showed a gradual and significant increase from M0 to M5, reaching 10.29 ± 1.61 g/dL (p=0.003), with minimal changes observed at M1, M2, and M3, and a more noticeable rise between M3 and M4. Ferritin levels displayed a consistent yet non-significant upward trend from M0 (64.45 ± 37.41 ng/mL) to M5 (92.90 ± 64.60 ng/mL) (p=0.399), while TSAT increased from M0 (12.89 ± 9.3%) to M5 (14.19 ± 5.12%), approaching but not reaching statistical significance (p=0.057). In the IV iron treatment group (Group IV), Hb levels consistently and significantly increased from M0 to M5, plateauing at 11.50 ± 0.96 g/dL (p=0.049). Ferritin levels rose significantly from M0 (60.71 ± 35.89 ng/mL) to M5 (289.42 ± 103.13 ng/mL) (p=0.0001), and TSAT levels increased significantly from M0 (11.33 ± 3.05%) to M5 (17.00 ± 6.92%) (p=0.031). These changes are illustrated in Figure 2. Throughout the study, albumin and hsCRP levels remained stable.

Data after drug withdrawal

In the post-treatment follow-up period at M4 and M5, distinct alterations in key biochemical markers were evident in both the OS and IV groups. At M4 and M5, the oral iron (Group OS) cohort displayed a stable trend in Hb levels, showing minimal deviation. Similarly, the IV iron (Group IV) treatment group maintained steady Hb levels from M4 to M5. Ferritin levels demonstrated a marginal change in the oral iron group, remaining relatively stable with little deviation between M4 and M5. In contrast, the IV iron group exhibited a consistent increase in ferritin levels from M4 to M5. Both treatment groups displayed fluctuations in TSAT levels. In the oral iron group, TSAT decreased from M4 to M5, while the IV iron group demonstrated stability in TSAT over the same period.

Adverse effects

In Group IV, which received IV iron, hypotension was the most prevalent adverse event, affecting four patients (30.7%). This was followed by infusion site reactions in three patients (23%) and headaches, also in three patients (23%). In contrast, Group OS, treated with oral iron, reported constipation as the most frequent adverse event in four patients (28.4%), followed by abdominal pain in three patients (21.4%) and headaches in two patients (14.2%). Notably, diarrhea and vertigo were absent in Group IV, whereas they were present in Group OS. A summary of adverse events experienced by patients in both treatment groups is provided in Table 6.

**Table 6:** Adverse events in the OS and IV groups

Adverse Event, n (%)	Oral Iron Group (N=14)	IV Iron Group (N=13)
Constipation	4 (28.4%)	2 (15.3%)
Diarrhea	1 (7.1%)	0 (0)
Abdominal pain	3 (21.4%)	0 (0)
Infusion site reaction	0 (0)	3 (23%)
Headache	2 (14.2%)	3 (23%)
Vertigo	1 (7.1%)	0 (0)
Hypotension	1 (7.1%)	4 (30.7%)

## Discussion

Anemia is frequently associated with CKD, significantly impacting cardiovascular health and increasing hospitalization rates among these patients. Addressing CKD-related anemia is a crucial aspect of patient care, with the National Kidney Foundation Kidney Disease Outcome Quality Initiative guidelines advocating for oral or IV iron supplementation as essential in managing iron-deficient anemic CKD patients [4]. Previous research has highlighted the similar efficacy of oral and IV iron supplements in correcting iron deficiency anemia (IDA) associated with CKD [5,6].

Despite numerous studies investigating the effectiveness of IV iron therapy in rectifying iron deficiencies and managing anemia in non-dialysis CKD populations, there remains a notable gap in comparative analyses between IV and oral iron therapies. This gap contributes to ongoing confusion, with studies drawing disparate conclusions due to varied parameters such as initial Hb levels, study duration, participants' iron status, sample sizes, and differences in IV iron formulations [7-9].

Clinical evidence consistently supports the superiority of IV iron over oral iron therapy in hemodialysis-dependent CKD patients. The limited absorption and slow incorporation of orally administered iron into the red cell mass in hemodialysis patients, especially without epoetin therapy, highlight the practical advantages of IV iron administration. Unlike oral iron, IV iron ensures complete absorption and utilization, avoiding issues related to inadequate absorption due to chelation by intraluminal cations or interference by certain medications. Additionally, by bypassing the gastrointestinal tract, IV iron reduces the risk of adverse gastrointestinal symptoms associated with oral iron supplementation, enhancing patient tolerance and compliance [10,11].

Moreover, IV iron therapy offers logistical advantages, particularly in patients unable to maintain adequate iron stores with oral supplementation. Current guidelines recommend IV iron for patients who fail to achieve sufficient iron status with oral supplementation. Despite the potential inconvenience of IV administration, recent studies have demonstrated the feasibility and safety of rapid IV iron administration, particularly in outpatient settings: administering 200 mg of iron sucrose intravenously over five minutes has been shown to be well-tolerated and convenient, minimizing the challenges of IV iron therapy, especially in outpatient settings [12,13].

Despite the efficacy and advantages of IV iron therapy, it may not be suitable for all patients. The IV route can be inconvenient or uncomfortable, particularly with prolonged infusion durations or concerns about potential allergic reactions [14]. Alternatively, oral iron therapy, although less efficient and associated with gastrointestinal side effects, may be preferred due to its convenience and familiarity. Moreover, oral iron therapy typically results in lower overall costs compared to IV therapy, largely due to reduced hospitalizations and associated healthcare needs. Additionally, the risk of anaphylactic or hypersensitivity reactions, although rare, can deter patients from opting for IV iron therapy [15,16].

Studies highlighting the limitations of non-liposomal oral iron formulations have raised concerns about their effectiveness in replenishing iron stores due to challenges in absorption, gastrointestinal side effects, non-compliance, and the prevalent inflammation seen in ND-CKD patients [7,8,17]. However, the emergence of liposomal iron offers promise as a new frontier in oral iron formulations. Utilizing ferric pyrophosphate enclosed within a phospholipid and sucrose esters of the fatty acid membrane, this innovative technology bypasses direct gastrointestinal interaction, facilitating direct intestinal absorption. The specialized architecture of liposomal iron ensures uptake by specific M cells in the small intestine, enabling its transport to hepatocytes, where enzymatic breakdown releases the encapsulated iron for utilization [18].

Our primary objective in conducting this prospective randomized trial was to assess the impact of both IV and liposomal oral iron on Hb levels in non-dialysis CKD patients. We also aimed to compare their efficacy in correcting Hb levels, assess patients' iron status during treatment, and evaluate their tolerance to the therapy. To ensure the reliability of our findings and avoid masking potential benefits on Hb levels, we specifically targeted patients with stable PTH levels corresponding to their CKD stage, maintaining a balanced calcium-phosphate ratio without any signs of inflammation.

Our results suggest that oral liposomal iron is non-inferior to IV iron in increasing Hb levels, albeit with a slower and less pronounced rise. Notably, IV iron demonstrated superior efficacy in replenishing iron stores, as evidenced by the substantial and quicker increases in ferritin and TSAT. This reinforces the idea that while oral liposomal iron is a viable alternative for correcting anemia, particularly in non-dialysis CKD patients, IV iron showcases swifter and more robust iron store replenishment.

While oral liposomal iron appears non-inferior to IV iron in correcting anemia, it is important to consider the broader clinical context and potential limitations of oral iron therapy. Traditional oral iron formulations are associated with poor absorption, gastrointestinal side effects, and variable efficacy, particularly in CKD patients who may have impaired iron absorption due to underlying inflammation or comorbidities. In contrast, liposomal oral iron formulations have been developed to improve bioavailability and reduce gastrointestinal side effects, offering a more tolerable and effective alternative to traditional oral iron preparations [19].

Several formulations of IV iron are currently available for the treatment of IDA in patients with CKD. These formulations include ferric carboxymaltose (FCM), ferric gluconate (FG), ferumoxytol, iron sucrose (IS), ferric derisomaltose/iron isomaltoside, and low molecular weight iron dextran (LMW ID). Each formulation has its unique pharmacokinetic and safety profile, making them suitable for different clinical scenarios [20-22].

The choice of IS as the IV iron formulation for this study was influenced by multiple factors, including its proven efficacy and favorable safety profile. Comparative trials involving other IV iron formulations, such as ferumoxytol and FCM, have shown them to be similarly effective in increasing Hb levels, with comparable safety profiles [23]. Additionally, iron sucrose is noted for its high tolerability and low incidence of adverse events [24,25]. These characteristics, combined with its widespread clinical use and availability, made IS the preferred agent for our trial. Its prevalent use in treating iron deficiency anemia in CKD patients aligns well with the demographic focus of our study on non-dialysis CKD patients.

Our study results were assessed alongside findings from four additional studies to gain more comprehensive insights into managing iron deficiency anemia in CKD patients. These studies, conducted in Italy, India, and the United States, examined various treatment strategies, including oral liposomal iron, IV iron, and oral non-liposomal iron, considering geographical and clinical differences across different populations [26-29].

The trends identified in the follow-up data from these studies reveal notable similarities with our findings. Pisani’s research emphasizes that oral liposomal iron is a safe and effective option for correcting anemia in non-dialysis CKD patients but acknowledges that IV iron is superior in rapidly replenishing body iron stores and accelerating iron repletion [26]. This aligns with our study, which also demonstrates that IV iron is more effective for rapid iron repletion compared to oral alternatives.

Similarly, both our study and Qunibi's research confirm that IV iron leads to a more pronounced and rapid increase in Hb levels compared to oral iron formulations [27]. The Agrawal study further supports these findings by highlighting the particularly significant impact of IV iron in specific patient populations, such as those with CKD on dialysis, where it induces a faster and more substantial increase in Hb levels [28].

In contrast, Charytan's research indicates that CKD patients receiving IV iron experienced increases in both Hb and ferritin, while those on oral iron saw increases in Hb without corresponding increases in iron stores [29].

Our study presents several limitations that warrant careful consideration. The small sample size restricts the generalizability of our findings to the broader CKD population. Additionally, the highly selective nature of the patient groups may not fully reflect the diversity encountered in clinical practice. While this selection minimized confounding factors related to renal anemia, such as inflammation, it limited our ability to evaluate the efficacy of liposomal iron in inflammatory states, which is a significant consideration, as inflammation commonly impairs iron absorption and utilization in CKD patients. Furthermore, while a subgroup analysis by CKD stage would be insightful, given the small numbers within each stage group, such analysis would lack statistical robustness. This limitation is pertinent as oral bioavailability of treatments typically decreases with advancing CKD stages due to multifactorial influences. Therefore, these analyses would be more appropriately conducted within larger, more diverse cohorts.

Secondly, the study did not investigate the potential effects of oxidative stress between the two types of iron or their impact on eGFR. Previous studies have reported conflicting results regarding the influence of iron on renal function, with some small-scale investigations suggesting that IV iron therapy may adversely affect renal tubular function and increase proteinuria [30].

Thirdly, due to the short follow-up period, it is uncertain whether the beneficial effects of liposomal iron observed in this study will persist long-term and significantly impact CKD outcomes. Longer-duration trials involving more patients would be necessary to address this question.

Finally, it is worth noting that this study did not compare liposomal iron to other oral iron formulations. Such a comparison could provide valuable insights into the relative efficacy and safety of different iron supplementation strategies.

## Conclusions

The management of anemia in CKD requires careful consideration of several factors, including the method of iron administration and the choice of treatment regimen. Our study findings suggest that oral liposomal iron presents a feasible alternative for treating anemia in non-dialysis CKD patients. Although it may not achieve the rapid and substantial iron repletion observed with IV iron, its lower incidence of adverse events, greater convenience, and reduced cost make it an attractive option for initial anemia management in uncomplicated CKD cases. Additionally, using oral iron instead of IV iron in CKD patients helps preserve venous capital, as oral administration eliminates the need for venous access for IV infusions, thereby reducing the risk of vascular damage and protecting vein integrity over time.
